# Salivarian Trypanosomes Have Adopted Intricate Host-Pathogen Interaction Mechanisms That Ensure Survival in Plain Sight of the Adaptive Immune System

**DOI:** 10.3390/pathogens10060679

**Published:** 2021-05-31

**Authors:** Stefan Magez, Joar Esteban Pinto Torres, Seoyeon Oh, Magdalena Radwanska

**Affiliations:** 1 Department of Molecular Biotechnology, Envirinmental Technology and Food Technology, Ghent University Global Campus, Songdomunhwa-Ro 119-5, Yeonsu-Gu, Incheon 406-840, Korea; Seoyeon.Oh@ghent.ac.kr (S.O.); Magdalena.Radwanska@ghent.ac.kr (M.R.); 2 Department of Biochemistry and Microbiology, Universiteit Gent, Ledeganckstraat 35, 9000 Gent, Belgium; 3 Laboratory for Cellular and Molecular Immunology (CMIM), Vrije Universiteit Brussel, Pleinlaan 2, 1050 Brussel, Belgium; Joar.Pinto@vub.be; 4 Department of Biomedical Molecular Biology, Universiteit Gent, Technologiepark Zwijnaarde 71, 9052 Gent, Belgium

**Keywords:** trypanosomiasis, adaptive immunity, parasitemia control, infection

## Abstract

Salivarian trypanosomes are extracellular parasites affecting humans, livestock and game animals. *Trypanosoma brucei rhodesiense* and *Trypanosoma brucei gambiense* are human infective sub-species of *T. brucei* causing human African trypanosomiasis (HAT—sleeping sickness). The related *T. b. brucei* parasite lacks the resistance to survive in human serum, and only inflicts animal infections. Animal trypanosomiasis (AT) is not restricted to Africa, but is present on all continents. *T. congolense* and *T. vivax* are the most widespread pathogenic trypanosomes in sub-Saharan Africa. Through mechanical transmission, *T. vivax* has also been introduced into South America. *T. evansi* is a unique animal trypanosome that is found in vast territories around the world and can cause atypical human trypanosomiasis (aHT). All salivarian trypanosomes are well adapted to survival inside the host’s immune system. This is not a hostile environment for these parasites, but the place where they thrive. Here we provide an overview of the latest insights into the host-parasite interaction and the unique survival strategies that allow trypanosomes to outsmart the immune system. In addition, we review new developments in treatment and diagnosis as well as the issues that have hampered the development of field-applicable anti-trypanosome vaccines for the implementation of sustainable disease control.

## 1. Introduction

Trypanosomiasis is a general name for diseases caused by trypanosomes, which affect both humans and animals, hampering the socioeconomic development of numerous endemic countries. Trypanosomes are protozoan parasites mostly transmitted by blood-feeding vectors. For some trypanosomes, transmission requires that part of the life cycle is completed inside the tsetse fly. This is the case for all *T. brucei* subspecies and is also the most efficient mode of transmission for *T. congolense* and *T. vivax*. The latter can, however, also be passed through mechanical transmission, as is the case for *T. evansi*. A unique situation occurs in case of *T. equiperdum*, which is closely related to *T. evansi*, but sexually transmitted between equines and hence does not fit the sensu stricto definition of a salivarian trypanosome. There is only one salivarian trypanosome that is considered to be a true zoonotic parasite, i.e., *Trypanosoma brucei rhodesiense*. This East-African trypanosome has an extended mammalian host reservoir that includes both game and domestic animals [1,2,3]. This makes the full eradication of HAT (human African trypanosomiasis) nearly impossible [4]. *T. b. rhodesiense* causes an acute, most often deadly, form of HAT. Due to its high-virulence characteristics, this form of sleeping sickness only accounts for a mere 2% of the total number of HAT cases. This means that the *Trypanosoma brucei gambiense* parasite, occurring in West and Central Africa, is responsible for the remaining 98% of all HAT cases. This parasite induces a much more chronic infection. With the human population being the main *T. b. gambiense* reservoir, these infections should be considered as an anthroponosis, rather than a zoonosis. The elimination of *T. b. gambiense* HAT as a neglected disease threat to sub-Saharan Africa is set to be attained by 2030 [4]. *Trypanosoma evansi* is, in general, not considered to be a human parasite, although several human infections have been reported in recent years as ‘atypical’ human trypanosomiasis [5]. The main reservoir for this parasite consists of domestic and game animals, making it a potential zoonotic threat to a large group of humans that live mainly in rural Asian areas, where close contact with cattle, and in particular water buffalo, still occurs on a daily basis [6].

Salivarian trypanosomes evolved to survive in a mammalian blood and lymph environment. Hence, they acquired the capacity to escape various immune defense mechanisms. At the same time, the basic principles of biological evolution result in the fact that trypanosomes adapt to interactions with a host, avoiding collateral damage. Indeed, with the host surviving for a prolonged period of time, the parasite ensures maximal probability of transmission. This favorable relationship is seen in several trypanotolerant African mammal species [7]. Interestingly, salivarian trypanosomes remain extracellularly throughout their life cycle. The latter is different from most other protozoan parasites that ensure optimal survival by hiding inside host cells. This means that trypanosomes are continuously exposed to attacks by the host innate immune system, as well as the adaptive humoral immune system. To thrive in this environment, salivarian trypanosomes have acquired multiple evolutionary strategies to evade and even destroy the host immune system. If infections are allowed to go on for prolonged periods of time, they will result in the death of susceptible host animals [8]. It is, however, rare that animals will succumb to excessive parasitemia levels in the blood or lymph fluid. Most often, AAT-associated death is the result of uncontrolled opportunistic infections, metabolic disorders such as inflammatory cachexia, or even neurological complication, particularly in case of *T. equiperdum*. During HAT, crossing of the blood-brain barrier will lead to the initiation of a lethal neuropathogenic stage of infection, the so-called second stage of infection, and reason behind the name ‘sleeping sickness’ [9]. To understand the immunopathology of trypanosomiasis, most experimental studies have focused on mouse *T. b. brucei* infections. While this approach limits the operational risks for researchers, the working model has been shown to reflect the most basic characteristics of infections with *T. b. rhodesiense*, *T. b. gambiense* and *T. evansi*. One limitation in this case is that mouse infections do not naturally result in cerebral complications. A second limitation could be that most experimental mouse research is conducted with trypanosome stabilates that give ‘good’ infections under laboratory conditions. This means that work is being conducted with mouse-adapted parasites that might have acquired characteristics that are no longer reflecting the dominant features of non-adapted parasites. This is particularly the case for *T. vivax* research, where field isolates are normally not able to infect laboratory mice. Hence, virtually all published host-parasite interaction data in this case are derived from a single isolate that was adapted to laboratory rodents several decades ago, i.e., the Y468 clone that originated from a field sampling in Nigeria [10].

While preventive vaccination for trypanosomiasis would be the only sustainable way to bring both HAT and AAT under control on a worldwide scale, no such approach exists today [11]. For this reason, disease control relies on the screening and diagnosis of patients, in combination with treatment. Vector control has been added to this strategy in several geographic locations, but this can only be successful when the vector range and reservoir is limited [12,13]. As failed anti-trypanosome vaccine experiments have difficulty passing through the traditional peer-review publication pipeline, conclusions about the reasons behind the lack of any successful strategy have to be deduced from the mere lack of publications showing the translation of so-called promising laboratory results into real field applications [8]. In contrast, there are multiple data available showing that trypanosomiasis, in particular in case of *T. evansi*, results in a general state of immunosuppression as well as the abrogation of commercial veterinary vaccine efficacy, and this for vaccines that are totally unrelated to the trypanosome infection itself [14]. All these effects are related to the general detrimental activity of trypanosomes on the host B and T cell compartment, an activity that is part of the parasite defense against the host antibody immune system.

## 2. The Life Cycle of Trypanosomes

**Figure 1:**
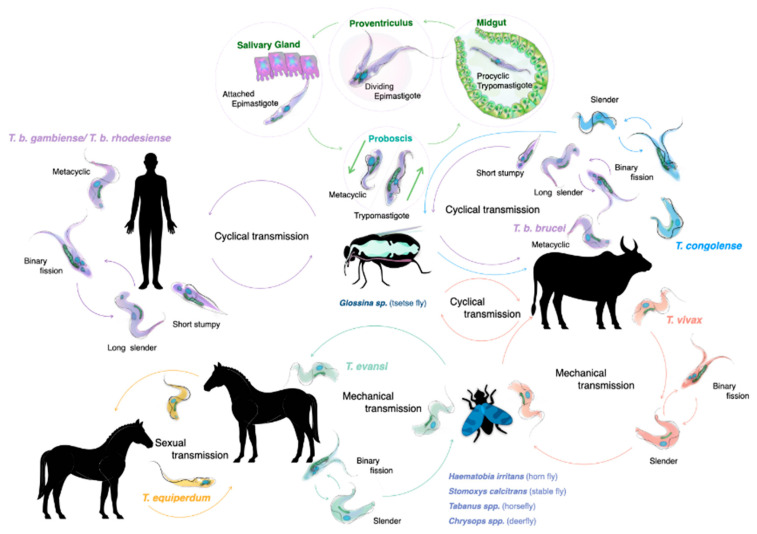
The life cycles of salivarian trypanosomes. For most HAT- and AT-associated trypanosomes, the tsetse (fly) serves as a central transmission vector. Most of the developmental stages of the trypanosome, as well as occasional sexual reproduction, take place inside the fly, making it the definitive host for the trypanosome. This is the case for all *T. brucei* sub-species, *T. congolense* and *T. vivax*. While *T. vivax* is also passed through mechanical transmission, involving mostly non-tsetse biting flies, such transmission is much less effective in the case of *T. congolense*. *T. evansi* is mainly transmitted by mechanical transmission through a wide host reservoir, while the closely related *T. equiperdum* is a sexually remitted parasite of equines.

The life cycle of salivarian trypanosomes has to be split into two categories: one that requires a developmental stage in the definitive host, the tsetse vector, where sexual reproduction occasionally does occur, and the other in which transmission occurs through mechanical passage, through contaminated mouth parts of insects or other blood-consuming animals such as vampire bats. As virtually all human infections are the result of an infected tsetse bite, this transmission mode and the associated trypanosome life cycle has traditionally received the most attention. Both *T. b. rhodesiense* and *T. b. gambiense* need the African tsetse to complete their life cycle (genus Glossina, with ‘tsetse’ itself meaning ‘fly’ in the Tswana language of southern Africa) [15]. Alternation between two completely distinct host species requires that parasites undergo differentiation in the mammalian bloodstream, resulting in the presence of both long slender parasites (i.e., proliferative form), and the short stumpy parasites (i.e., the non-proliferative form). When taken up by the tsetse during a blood meal, it is the short stumpy form that allows the continuation of the life cycle. While confronting the digestive system of the fly, parasites must resist a strongly alkaline enzyme-rich environment. This is achieved by rapid differentiation into procyclic trypomastigotes, and subsequent multiplication by binary fission. After penetration of the peritrophic matrix that covers the gut epithelium, parasites migrate to the ventriculus where they transform into long and short epimastigotes through asymmetrical division. Short epimastigotes can migrate to the tsetse salivary glands, where they differentiate into infective metacyclic trypomastigotes that can undergo meiotic division [16]. The latter is not an obligatory step to complete the life cycle, but it allows the parasite to increase genetic variability [17,18]. The full tsetse cycle takes about three weeks to be completed [19] (Figure 1). Interestingly, saliva-stage parasites are able to decrease the tsetse feeding efficiency due to alterations in the salivary gland composition. Indeed, in non-infected flies, salivary anti-coagulation and anti-platelet aggregation activity ensures that blood flows unobstructed during feeding. These activities are suppressed in trypanosome-infected flies, hampering the feeding efficiency and increasing the feeding frequency. This increases parasite transmission chances [20]. Tsetse saliva also accelerates *T. brucei* infection by inhibiting bite site inflammation [21,22].

After successful transmission, metacyclic parasites that enter the mammalian blood circulation will use a surface glycoprotein called the metacyclic variant surface glycoprotein (mVSG) as a first defense against the host antibody attack [23]. However, as trypanosomes have a very limited repertoire of mVSG encoding genes, surface recognition by host antibodies will quickly improve. Therefore, prolonged survival requires a new adaption approach by the parasite and proliferating long slender bloodstream trypanosomes use a much wider range of bloodstream form VSGs [23]. As *T. brucei* parasites have access to a battery of more than 1000 VSG genes and pseudo-genes, expressed from approximately 15–20 expression sites, this strategy has been suggested to allow the trypanosome to outrun the host antibody response for an ‘eternal’ period of time [8,11,24]. One interesting issue here is that if the parasite would be ‘too’ successful in evading immune control, this would lead to the unfortunate death of its host. In order to avoid this, trypanosomes have developed quorum sensing mechanisms that have been studied best for *T. brucei*. This system regulates the transition from the dividing long slender bloodstream forms to the non-dividing short stumpy forms [25]. It involves the oligopeptidase transporter TbGPR89 as a ‘sensor’ for peptide breakdown products, an activity attributed to the action of proteases secreted by the parasite [26]. Besides limiting peak parasite levels, this system also prepares the parasite for transition to the tsetse vector [27]. Finally, quorum sensing most likely also monitors the inflammatory state of the host, contributing to parasitemia peak-height control in terms of host pathology development [28,29].

Compared to the available knowledge of the *T. brucei* life cycle, the cycle of other salivarian trypanosomes is less well documented [30,31,32]. Interestingly *T. congolense* is more effective in establishing tsetse infections compared to *T. brucei*, with the parasite being particularly effective in reaching the proboscis of the fly. Here, trypomastigote–epimastigote transformation occurs. Hence, while both *T. brucei* and *T. congolense* are transmitted through the same vector, there are differences in the way the two trypanosomes infect and occupy the body of the tsetse. It is possible that *T. brucei* adopted a survival strategy in the salivary gland, as this niche would not be occupied by the much more efficiently growing *T. congolense* parasites. Finally, meiotic reproduction in the tsetse vector has also been reported to occur in *T. congolense* [33].

**Figure 2:**
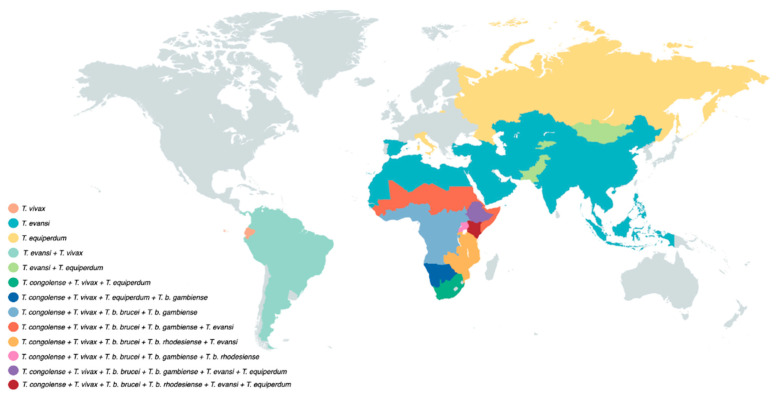
Salivarian trypanosomes have a vast near-worldwide distribution. Tsetse-transmitted *T. brucei* parasites occur only in sub-Saharan Africa, with the human-infective *T. b. gambiense* being present in West and Central Africa, while *T. b. rhodesiense* is restricted to East Africa. *T. congolense* has a similar sub-Saharan Africa distribution. Due to the possibility of mechanical transmission, *T. vivax* has a wider distribution and occurs in sub-Saharan Africa as well as South America. *T. evansi* has an even wider geographic distribution, including locations on four different continents. *T. equiperdum* has a rather unique distribution pattern as it does not use insect vector transmission as a means of propagation.

Because of its mechanical transmission, *T. evansi* has a much simplified life cycle. Here, the long slender morphology is the only form seen in the bloodstream of the mammalian host. In fact, it is accepted by many that *T. evansi* is a ‘variant’ of *T. brucei*, having lost the kinetoplast DNA (kDNA), which is essential for development in the gut of the tsetse fly [34,35]. One could assume that the loss of the capacity to infect the tsetse vector would have resulted in a detrimental evolutionary step for the trypanosome, but that is obviously not the case. Instead, *T. evansi* very efficiently relies on fly-free transmission. Indeed, non-tsetse mediated spread has allowed the parasite to move and be transported to most parts the world, aided by the fact that many infected animals hardly show any symptoms [36]. *T. evansi* is now found in various northern and southern regions of Africa, South and Central America, the Middle East, China, the Indian subcontinent, Southeast Asia, parts of Oceania and occasionally even in Europe [6,37,38,39] (Figure 2). 

The main host reservoir depends mostly on local agriculture conditions and includes horses, camels and buffaloes. Wildlife such as capybaras and deer can also serve as the host reservoir as well as cattle, pigs, goats and dogs [36,37]. Of note is that its global distribution allowed *T. evansi* to be historically discovered as the first pathogenic trypanosome, responsible for the animal disease ‘surra’ in India [37]. This ‘first’ discovery has more recently triggered a debate on nomenclature of salivarian trypanosomes as a whole [40]. 

## 3. Trypanosomiasis and the Human Biochemical Defense System

*T. b. rhodesiense* HAT is a rare disease that recently has only been reported in six East African countries [4,41]. However, there is a general impression that current local cases are being underreported as *T. b. rhodesiense* HAT accounts for two-thirds of all tourist HAT cases [42]. *T. b. gambiense* HAT was still reported in 15 sub-Saharan countries in 2018 [4,41] (Figure 2). HAT infections are characterized by a first hemolymphatic phase, with parasites invading the host’s circulatory and lymphatic systems, causing immune dysfunction. Initial infection is mainly characterized by fever, weakness, enlarged lymph nodes and joint pains. Once the parasite passes through the blood-brain barrier, the disease enters the meningo–encephalitic ‘second stage’, causing neuropsychiatric symptoms such as daytime sleepiness and nocturnal insomnia as a result of the fragmentation of the circadian rhythm [43,44]. These symptoms precede the death of the victim, if left untreated. Symptoms of both *T. b. rhodesiense* and *T. b. gambiense* HAT are very similar, with the main difference being that it generally takes much longer for the disease to progress into the second stage in cases of *T. b. gambiense* HAT. As already outlined, humans are resistant to *T. b. brucei*, *T. congolense* and *T. vivax*, and in most cases even *T. evansi*. This is due to an intrinsic ‘innate’ biochemical resistance that is present in human serum (as well as the serum of gorillas and certain old-world monkeys such as baboons). This activity is embodied by two factors called trypanolytic factor 1 (TLF1) and TLF2 [45,46]. Both factors are high-density lipoprotein complexes containing apolipoprotein A1, the primate-specific ion channel-forming protein apolipoprotein L-1 (APOL1) and the hemoglobin binding protein haptoglobin-related protein (HPR) [47,48,49]. TLF2 contains additional IgM molecules [45,50]. Although activity of TLF2 has long been known to be the major trypanolytic factor [51,52], TLF1 is the better studied factor due to the relative ease of purification. The functional mechanisms of both TLF1 lysis of trypanosomes in general, and the resistance of human infective trypanosomes, has been most rigorously studied in a *T. b. brucei/T. b. rhodesiense* comparison, despite *T. b. gambiense* HAT being obviously the most important problem for human health. This is because the resistance mechanism of T. b. gambiense is more complex and diverse, as outlined below. The main idea behind this innate defense interaction is the problem the trypanosomes face during the rapid proliferation phase, i.e., the need for uptake of host iron. This is ensured by the surface expression of a specific heterodimer surface receptor consisting of the VSG-related molecules ESAG6 and ESAG7 [53,54]. However, trypanosomes acquire additional iron through the scavenging of heme groups, abundantly available as part of hemoglobin. This hemoglobin is often bound to other compounds, forming complexes such as TLF1. In *T. brucei*, uptake of TLF1 is mediated by the specific receptor TbHpHbR (haptoglobin–hemoglobin receptor) [55,56]. TLF2 uptake largely occurs independent of the TbHpHbR receptor [57], but involves IgM-mediated uptake [50]. In both cases, the central role of APOL1 is crucial for the trypanosome membrane disruptions induced by normal human serum (NHS) [58,59]. Interestingly, baboon APOL1 is much more potent than human APOL1. This results in the fact that baboon serum confers resistance not only against non-human infective trypanosomes, but also the human infective trypanosomes causing HAT [60,61]. As *T. b. rhodesiense* is a human pathogen, it is resistant to the lytic action of APOL1. This property is linked to the expression of the serum resistance-associated (SRA) protein, a molecule that can physically block the formation of the pore-forming conformation of APLO1 inside the endocytic pathway of the parasite [62,63,64,65]. To understand the NHS resistance of *T. b. gambiense*, it should first be noted that this is not a homogenous family of parasites, but is separated into two groups. Group 1 *T. b. gambiense* parasites exhibit consistent NHS resistance, show little genetic variation within a given geographic location, and are characterized by the genetic marker TgsGP [66]. Group 2 *T. b. gambiense* parasites are a much more heterogeneous group of organisms lacking a specific marker, showing variable NHS resistance, being much closer related to *T. b. rhodesiense* and *T. b. brucei*, and representing the zoonotic side of *T. b. gambiense* HAT [5]. NHS resistance of *T. b. gambiense* has so far mainly been studied in terms of TLF1 activity, with the Group 1 parasites exhibiting a reduced uptake of the complex [67], linked to reduced expression and mutation of the HpHb-receptor [56,68,69,70]. While the TgsGP molecule further improves APOL1 resistance by reducing trypanosomal membrane fluidity [71,72], a cysteine protease has been identified as a third factor contributing to Group 1 *T. b. gambiense* NHS resistance [72]. Group 2 *T. b. gambiense* parasites show a variable degree of resistance that is independent of TLF1 uptake [73]. In addition, with TgsGP not being universally present in Group 2 parasites, and no information being available with respect to the exact nature of the cysteine protease activity involvement in APOL1 resistance, it is not clear if this mechanism is active in Group 2 *T. b. gambiense* either. Given the dearth of data that could universally explain the TLF1 resistance of *T. b. gambiense* parasites, combined with the lack of any functional data on TLF2 resistance, it is clear that the explanation of the true nature of *T. b. gambiense* resistance is still awaiting full elucidation [74].

As already outlined above, *T. evansi* is a mechanically transmitted animal parasite that has the widest geographic distribution range of all salivarian trypanosomes (Figure 2). Whether or not the infection can be considered as a zoonosis threat is a matter of debate. There have been several case reports of atypical *T. evansi* human trypanosomiasis. In none of these cases is it clear how transmission occurred, although all infections occurred in the vicinity of infected livestock [75]. When the first aHT case was reported in India, susceptibility of the patient coincided with a mutation at the level of the APOL1 gene, possibly explaining the lack of NHS trypanolytic activity [76]. A second case in Vietnam, however, occurred in a patient with functional copies of the APOL1 gene, and normal serum APOL1 levels [77]. This indicates that the true mechanism by which *T. evansi* parasites have acquired a serum resistance mechanism still needs to be elucidated, or that different parasites have acquired different mechanisms, similarly to the situation outlined above with Group 2 *T. b. gambiense*. The latter notion could be supported by the fact that that *T. evansi* parasites are actually a group of heterogeneous parasites with multiple independent origins. They are often closely related to *T. brucei* parasites found in the same geographic regions, and only distantly related to other *T. evansi* parasites found in more remote locations [78,79]. Hence, more effort is required to fully understand the nature of the trypanosome–host interplay during aHT.

## 4. Innate and Adaptive Immunity to Trypanosomiasis

**Figure 3:**
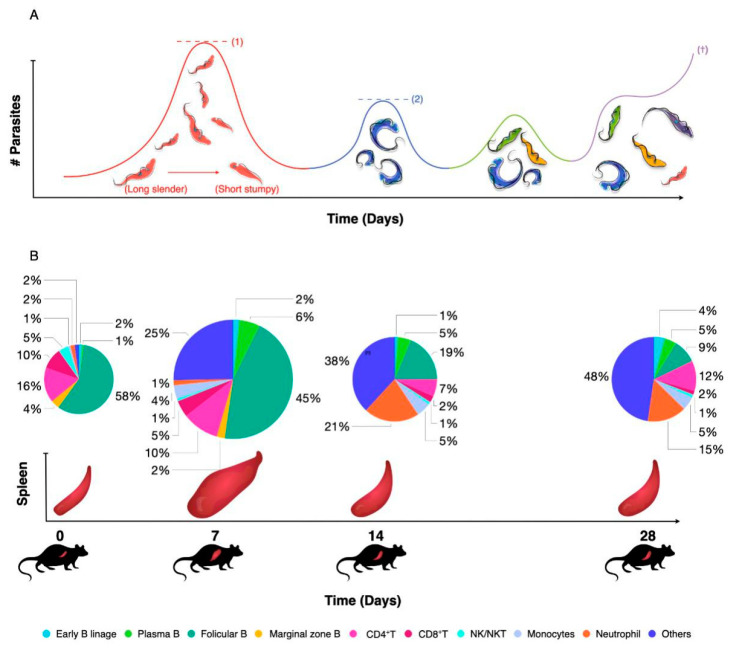
(**A**) Salivarian trypanosomes use antigenic variation of their surface coat as a first line of defense against host antibody attack. During early infection, quorum sensing ensures that peak parasitemia does not reach lethal levels (1). After clearance of the first variant, parasitemia is characterized by the presence of parasites expressing a novel VSG coat, usually giving rise to several low-peak infections (2). Improved peak control results from a combination of antibody activity, innate inflammatory responses and intrinsic quorum sensing. Subsequent parasitemia waves start to be comprised of multiple VSG variants that occur at the same time, indicating a loss of proper antibody-mediated parasite population control. In experimental models, infection will most often result in late-stage uncontrolled parasitemia and death (†). (**B**) As early parasitemia progresses in mice, infection-associated splenomegaly results in an initial increase in organ size and cellularity (7 dpi). By 14 dpi, spleen cell numbers usually drop and important populations such as Marginal Zone B cells start to disappear. Organ structure is also completely destroyed. As infection progresses, most adaptive immune cell populations collapse, while the spleen is being filled with non-immune cells such as pre-erythrocytes. This stage of spleen dysfunction coincides with the loss of parasitemia control. The diameter of the pie-charts is representative of the total spleen numbers during infection. Percentages of all major immune cell populations are indicated in the color-coded pie charts.

The impact of trypanosomiasis on the host innate and adaptive immune response has recently been reviewed in great detail at the level of both B and T cell biology and with a link to inflammatory macrophage biology [8]. As already outlined above, trypanosomes have adopted a system of antigenic coat variation to escape from the antibody immune system [24]. The surface expression of a dense layer of VSGs is crucial here, as (i) it allows regular escape from antibody attacks through epitope variation, (ii) it provides for antibody surface clearance through lateral movement of VSG-antibody complexes towards the flagellar pocket, where endocytosis results in surface ‘cleaning’ [80], (iii) it constitutes a physical defense barrier, making complement-mediated attacks nearly irrelevant for parasitemia control [81] as well as a scavenger system to prevent complement surface fixation by VSG shedding [82], and (iv) it serves as a highly immunogenic decoy and immunomodulatory interaction surface with the immune system that ultimately seems to deregulate the immune system in favor of the parasite. The latter starts with the inflammatory properties of the VSG–GPI anchor itself [83], driving early infection in the host towards the production of IFNγ and TNF [84,85,86], coinciding with both macrophage and neutrophil activation in vital organs such as the spleen and the liver [87,88,89]. While this might help the host to control parasitemia through multiple immune mechanisms such as parasite phagocytosis and parasite growth control [90,91], it also drives deregulation and destruction of the host B cell compartment [92]. Finally, these infection-induced immune complications result in a failure of anti-VSG recall responses as well as a failure of other memory B cell responses [29,93]. The latter could be considered as collateral damage, but the loss of anti-VSG memory by the host means that ‘old’ or ‘previously used’ VSG molecules can be reused later on in infection. In addition, newly arising mosaic VSG variants that can carry cross-reactive epitopes can also be expressed on the surface as fully functional VSG coats [94] (Figure 3). Deregulation of the B cell compartment by the trypanosome also requires a parasite intervention at the T cell level, as these cells play a crucial role in the resistance to trypanosomiasis [95]. Indeed, even if antigenic variation were to be fully efficient at the level of the surface-exposed VSG B cell epitopes, it would only partially evade the efficiency of the hosts’ B cell immunity. That is because more structurally conserved cryptic epitopes of the VSG will trigger the build-up of T cell memory. Hence, persistent T cell help would readily be available for any newly arising B cells against any newly arising VSG variant, making use of T cell receptor recognition of conserved VSG T cell epitopes presented by the B cell MHCs [96,97]. To avoid this from happening, trypanosomes have adopted mechanisms of T cell suppression [98,99].

## 5. Recent Advances in the Diagnosis of HAT and AT

As the clinical signs of trypanosomiasis are unspecific, the ‘only’ accepted way of confirmed diagnosis before treatment, especially in case of human infections, is by microscopic identification of the parasite. Based on general symptoms such as fever, anemia, and hemodilution, HAT patients are often misdiagnosed as having malaria. However, correct early diagnosis is essential for successful treatment. To improve microscopy detection, blood analysis can be performed on the buffy coat [100]. Mini anion exchange chromatography (mAECT) can be used to eluate parasites from blood samples, prior to microscopy analysis [101,102]. Fluorescent dyes that intercalate nucleic acids have been shown to improve the microscopy detection limit, but are not often used under field conditions [103]. When parasites cannot be detected in the blood, microscopy analysis of aspirate fluid from swollen cervical lymph nodes can be used as an alternative method, while cerebrospinal fluid can be analyzed to confirm the neurological state of the infection [42,104]. One main issue with all the techniques mentioned here is that they are all extremely labor-intensive when disease prevalence is low. For this reason, a number of pre-screening methods have been developed, aiming at excluding true negative individuals. Implementation of the card agglutination test (CATT) for the detection of *T. b. gambiense* more than 40 years ago was a major breakthrough [105]. A similar test exists for *T. evansi* [106]. Unfortunately, no equivalent test exists for the detection of *T. b. rhodesiense*. CATT is based on the detection of antibodies that cross-react with particular VSG molecules, and has a high negative predictive value (NPV) as well as high sensitivity and specificity. However, the test has a relatively low positive predictive value (PPV), meaning that all CATT-positive individuals require a parasitological screening, a technique that is time consuming and requires a skilled analyst [107]. When a CATT-positive score is confirmed by microscopy, patients undergo a ‘staging’ screening through a cerebrospinal fluid analysis. This invasive technique is absolutely required for the correct choice of treatment [104].

**Figure 4:**
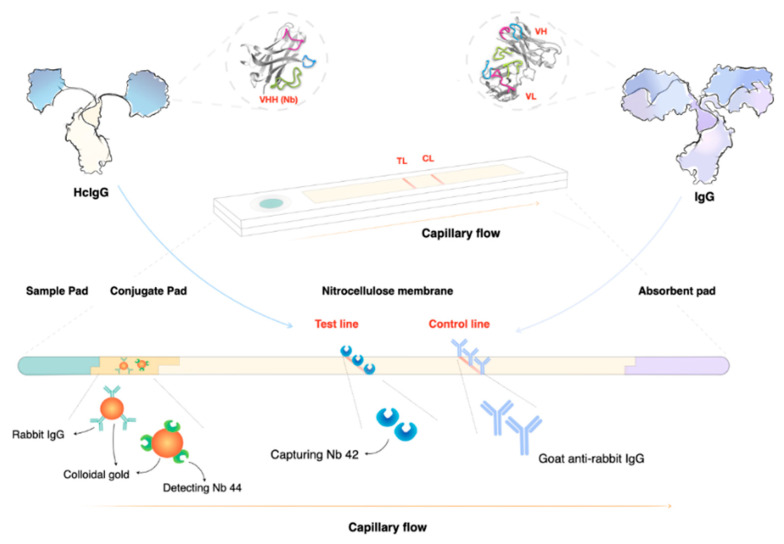
Lateral flow assays (LFAs) are ideal as point-of-care (POC) tools. In the case of nanobody-based LFAs, the test line consists of a printed line of highly specific nanobodies (Nbs) that can capture their target even in the presence of host antibodies that bind the same antigen (Ag). This can be achieved due to the unique nature of heavy-chain camelid antibodies (HcIgG) that bind their target in the absence of the light chain that is present in conventional antibodies (IgG). Detection of a parasite Ag can be done using a gold-conjugated second sandwich Nb that is pre-incubated on the conjugation pad. At the point of sample application, the detection Nb will bind the target, and together they will migrate towards the printed capturing line. Sandwich formation and Ag accumulation will result in the development of a red line. A second control line is used to ensure the correct interpretation of the test results. Ag-detecting LFAs can be used as proof of infection, as well as a test of cure. This makes the format unique compared to antibody detecting LFAs, which measure ‘exposure’ rather than active infection.

In recent years, multiple efforts have been undertaken to transform CATT into a more user-friendly lateral flow format that can be used as a point-of-care (POC) diagnostic tool [108,109,110,111,112]. Important to note is that all these tests are based on antibody detection, which is a measurement of exposure, and not infection. As such, it is unlikely that any of these approaches will have a drastically improved PPV. Hence, today there is still a need for the implementation of diagnostic tools that can detect the parasite, or components released/secreted by the parasite. While PCR is obviously suitable for direct pathogen detection, this technique has limitations in resource-poor field POC settings. Here, loop-mediated isothermal amplification (LAMP) technology appears to be easier to implement [113,114,115]. The development of easy-to-use high-PPV diagnostic tools for trypanosomiasis is crucial, especially now that disease prevalence is in decline. A second factor that has to be taken into account is that when the human reservoir is being controlled, the relative importance of zoonotic transmission increases. Hence, it is clear that large herd screenings of asymptomatic animals that serve as an everlasting reservoir for human infective parasites will become more important [116]. This means that in order to implement a sustainable control of worldwide trypanosomiasis, the development of tools for the detection of animal trypanosomiasis needs to receive more attention. In this context, the targeting of *T. evansi* is of utmost importance. So far, diagnosis of this parasite has heavily relied on the detection of one specific VSG, i.e., the very common RoTat1.2 VSG, by either antibody detection or by molecular biology methods [106,117]. Unfortunately, this makes the test unsuitable for *T. evansi* detection in regions where RoTat1.2-negative *T. evansi* Type A or B parasites occur [78,118]. For the detection of *T. evansi* Type B, a highly sensitive LAMP assay has been developed [119]. One of the most recent developments for the detection of *T. evansi* is the implementation of recombinase polymerase amplification (RPA) combined with lateral flow detection [120]. Here, the detection of *T. evansi* is achieved through isothermal DNA amplification at 39 °C, resulting in an easy-format readout within 20 min. Finally, also for the detection of *T. congolense*, a rapid diagnostic POC tool has been developed, this time based on nanobody technology [121]. This assay detects pyruvate kinase that is secreted by metabolically active trypanosomes and hence can be used as a high PPV test for the detection of active parasitemia, and as a test-of-cure after anti-trypanosome drug therapy [121]. Active trypanosome case detection using nanobody-based technology has also been proposed by targeting the secreted *T. congolense* glycolytic enzyme aldolase [122,123,124], as well as the *T. evansi*-secreted enzyme enolase [125]. In all these settings, nanobodies derived from single-chain camelid antibodies have proven to be successful in binding target epitopes that remain accessible even in the presence of an anti-parasite immune response. Due to the unique configuration of nanobodies, combined with their small size, they can avoid epitope binding competition with infection-induced host antibodies (Figure 4).

## 6. Recent Advances in Treatment of HAT

For nearly a century, treatment of HAT has relied on a very limited set of drugs that all have a string of severe negative side effects. These include pentamidine for the treatment of first-stage *T. b. gambiense* HAT, and nifurtimox or eflornithine for the treatment of second-stage *T. b. gambiense* HAT. Suramin as well as melarsoprol have been used for treatment of first-stage *T. b. rhodesiense* HAT. Melarsoprol, however, being an arsenical compound, shows extreme high toxicity and severe side effects, including reactive encephalopathy as a major fatal outcome in up to 10% of patients. Hence, in optimal circumstances, this drug should be restricted in its use for treatment of second-stage *T. b. rhodesiense* infection only [126]. In 2009, a new drug regimen for the treatment of second-stage *T. b. gambiense* HAT was proposed, using a combination of nifurtimox and eflornithine (NECT) [127]. This mixed therapy reduces the complexity of the previously used eflornithine therapy. Both drugs are provided free of charge by the WHO to endemic countries, with a kit containing all the material needed for administration. Most recently, in 2018, fexinidazole has been made available as an oral therapy for *T. b. gambiense* HAT and has been incorporated in the WHO interim guidelines as one of the first-line treatments for HAT [128]. The drug can also be used to cure non-severe second-stage patients [129].

Treatment of *T. congolense* and *T. vivax* animal trypanosomiasis relies in large on the use of diminazene diaceturate, isometamidium and, unfortunately, homidium (ethidium bromide) [130]. While diminazene diaceturate can also be used effectively for the treatment of *T. evansi* infections, it has not been registered for use in humans, not even for aHT, due to the severe side effects in the treatment in animals, including dogs [131]. Diminazene cannot cross the blood-brain barrier, and therefore it is not effective in the case of central nervous system infections [130]. In the absence of systematic data on aHT caused by *T. evansi*, there has been no registered treatment strategy for this disease to date. However, in the cases of aHT outlined above, a successful cure was obtained after treating with suramin [75].

## 7. The Lack of Anti-Trypanosome Vaccination Still Hampers Sustainable Disease Control

There is no vaccine strategy available for the prevention of either human or animal trypanosomiasis. Early on in trypanosome immunology research, it was discovered that the dense surface presence of the VSG, together with the inexhaustible gene repertoire of VSG-encoding genes, allows the parasite to escape from any major antibody attack [24]. However, in between the VSGs, there are a number of invariant surface glycoproteins present that have been the target of several alternative vaccination approaches, although none of these have yet resulted in any field application [11]. A major hurdle in the development of anti-trypanosome vaccines is that salivarian trypanosomes have acquired the ability to cause significant and permanent damage to the mammalian humoral immune system. In experimental *T. brucei* models, this has been shown to result in the non-specific loss of vaccine-induced B cell memory responses as well as T cell memory [93,132]. It is not clear whether or not this pathology affects human infections, as this has not been properly addressed by any field study. The only data available to date relates to the fact that *T. b. gambiense* HAT results in the significant reduction of anti-measles host antibodies in individuals vaccinated against this non-related infectious disease. Upon recovery after HAT treatment, these anti-measles antibody titers remained low, but above the theoretical threshold considered ‘protective’ in the test used [133]. Whether or not this assessment is correct has not been verified, but given the previously published warning that HAT results in false-positive scores in HIV antibody diagnostic tests [134], it could well be that the detrimental effect of HAT on human vaccine memory is greater than so far reported. However, based on experimental mouse studies, it can be anticipated that the damage done to the human immune system would be far greater in infections with high parasitemia levels, i.e., *T. b. rhodesiense* infection. Unfortunately, no human data is available from which to confer the validity of the observations obtained in virulent mouse trypanosome infection models. As for the negative impact of *T. evansi* on the mammalian immune memory compartment, all data available is derived from animal infection models. Here it has been shown that the parasite undermines memory responses of several non-related vaccines [14,135,136]. Hence, it could be expected that also in atypical *T. evansi* human trypanosomiasis, the destruction of the host B cell compartment could be one of the detrimental outcomes of infection. With respect to *T. congolense* and *T. vivax* infections, field data on the detrimental effect of infection on vaccine-induced memory is lacking, but experimental models for both infections showed the same severe detrimental impact on the host B cell compartment [94,95].

## 8. Conclusions

In the past 10–15 years, a tremendous effort has been made to bring *T. b. gambiense* HAT under control and eliminate the public health threat of the disease in sub-Saharan Africa. This achievement is the result of an international collaboration not just between affected countries, but also with research communities within universities, partner organizations such as DNDi (Drugs for Neglected Diseases initiative), FIND (Foundation for Innovative New Diagnostics), BMGF (Bill & Melinda Gates Foundation) and numerous national research and grant-providing organizations from European countries and the USA. If efforts are sustained, it appears that transmission of Group 1 *T. b. gambiense* HAT can be brought to a minimum by 2030. However, it would be a serious mistake to assume that this is the end of HAT as a disease. Indeed, neither *T. b. rhodesiense* nor Group 2 *T. b. gambiense* HAT have received the same level of attention, and the zoonotic nature of these infections makes them much harder to control. Control of *T. congolense* AAT and *T. vivax* AT is even further from being realized. Finally, while *T. evansi* AT is the most widespread form of animal trypanosomiasis, spanning five continents, the aHT disease variant is rare and as such has not been receiving any serious attention. However, with the ever-increasing geographic presence of *T. evansi*, this form of trypanosomiasis could increase in the future, unless its animal reservoir is being tackled in a systematic manner. Without the availability of a field-applicable anti-trypanosome vaccine, this will be a very arduous task that will require continued dedication and international partnerships between countries where the disease is endemic and countries at risk of importing the disease through traffic of seemingly healthy, but infected, animals.
